# Initial experience with a dedicated cardiac MRI program for congenital heart disease in a limited resource environment

**DOI:** 10.1186/1532-429X-15-S1-P290

**Published:** 2013-01-30

**Authors:** Mahesh Kappanayil, Ramiah Rajeshkannan, Ashish Sapre, Krishna Kumar

**Affiliations:** 1 Pediatric Cardiology, Amrita Institute of Medical Sciences and Research Centre, Kochi, India; 2 Radiology, Amrita Institute of Medical Sciences and Research Centre, Kochi, India

## Background

Populous developing nations like India have a huge disease burden of CHD, many of the patients being older, with uncorrected or partially palliated complex lesions. Despite its powerful diagnostic advantages in this specific clinical milieu, cardiac MRI is yet to find widespread acceptance , the reasons being poor awareness regarding CMR and its clinical utility, infrastructural costs and lack of trained personnel.

## Methods

Dedicated CMR unit for CHD was established at our tertiary cardiac care centre in South India in January 2010 comprising 1.5 Tesla GE SIGNA HDxt scanner & dedicated personnel (Pediatric Cardiologist and Cardiac Radiologist). All patients undergoing CMR were prospectively included in a database.

## Results

175 patients; median age: 11 years (1year-65years) underwent CHD-CMR (January 2010 - September 2012). Thirty required general anesthesia. The most common indication (in 64%) was comprehensive anatomic/physiological evaluation for unoperated or palliated complex CHD, including complex double-outlet right ventricle (n=30), L-TGA (n=14), complex heterotaxy syndromes (n=7) and single ventricles (n=8). 33% were ≥10 years age. Protocols for sequences and imaging planes were customized according to information desired. Accurate anatomical information (intracardiac and extracardiac) was obtained in all. In 10%, new anatomic details (not identified by other modalities) were seen. Physiological information obtained included ventricular function, shunt ratios, and blood flow dynamics. Jugular venous pressure invasively was recorded for post Glenn shunt patients undergoing CMR. Data withstood internal cross-validation in 97%. Additional imaging was requested-for in 6 patients after CMR.

23% underwent cardiac surgery on basis of information obtained/augmented by CMR - including double-switch (Senning + Rastelli), complex primary 2-ventricle repairs for DORV, complex biventricular conversion following previous single ventricle palliation, conduit placement/replacement , Bentall operation and Fontan. Complex repairs were significantly aided by CMR in terms of both decision-making and surgical planning.

Challenges included need for specialized training, learning curve influenced by a unique patient-subset, relatively long procedure (45 - 90 min) and post-processing time, logistics of sharing MRI scanner with radiology, poor initial acceptance by professional colleagues, and financial considerations.

## Conclusions

It is feasible to establish dedicated CHD-CMR services, even in resource-limited, developing country environments. It may in fact have special value in this unique patient milieu comprised predominantly of older patients with complex lesions.

Availability of CMR has the potential to positively impact the development and maturation of surgical practices in selected CHD programs in developing nations.

CHD-CMR units in developing world can form the new research hubs for further technological developments in the field.

## Funding

Nil

